# Review: A Contemporary, Multifaced Insight into Psoriasis Pathogenesis

**DOI:** 10.3390/jpm14050535

**Published:** 2024-05-16

**Authors:** Rucsandra Cristina Dascălu, Andreea Lili Bărbulescu, Loredana Elena Stoica, Ștefan Cristian Dinescu, Cristina Elena Biță, Horațiu Valeriu Popoviciu, Răzvan Adrian Ionescu, Florentin Ananu Vreju

**Affiliations:** 1 Department of Rheumatology, University of Medicine and Pharmacy of Craiova, 200349 Craiova, Romania; rucsandrag@gmail.com (R.C.D.); cristina.gofita@umfcv.ro (C.E.B.); florentin.vreju@umfcv.ro (F.A.V.); 2 Department of Pharmacology, Faculty of Medicine, University of Medicine and Pharmacy of Craiova, 200349 Craiova, Romania; 3 Department of Dermatology, Faculty of Medicine, University of Medicine and Pharmacy of Craiova, 200349 Craiova, Romania; loredana.stoica@umfcv.ro; 4 Department of Rheumatology, BFK and Medical Rehabilitation, University of Medicine, Pharmacy, Science and Technology of Targu Mures, 540139 Mures, Romania; horatiu.popoviciu@umfst.ro; 5 Third Internal Medicine Department, ‘Carol Davila’ University of Medicine and Pharmacy, 020021 Bucharest, Romania; razvan.ionescu@umfcd.ro

**Keywords:** psoriasis, microbiome, genetics, cytokines, signaling pathways

## Abstract

Psoriasis is a chronic recurrent inflammatory autoimmune pathology with a significant genetic component and several interferences of immunological cells and their cytokines. The complex orchestration of psoriasis pathogenesis is related to the synergic effect of immune cells, polygenic alterations, autoantigens, and several other external factors. The major act of the IL-23/IL-17 axis, strongly influencing the inflammatory pattern established during the disease activity, is visible as a continuous perpetuation of the pro-inflammatory response and keratinocyte activation and proliferation, leading to the development of psoriatic lesions. Genome-wide association studies (GWASs) offer a better view of psoriasis pathogenic pathways, with approximately one-third of psoriasis’s genetic impact on psoriasis development associated with the MHC region, with genetic loci located on chromosome 6. The most eloquent genetic factor of psoriasis, PSORS1, was identified in the MHC I site. Among the several factors involved in its complex etiology, dysbiosis, due to genetic or external stimulus, induces a burst of pro-inflammatory consequences; both the cutaneous and gut microbiome get involved in the psoriasis pathogenic process. Cutting-edge research studies and comprehensive insights into psoriasis pathogenesis, fostering novel genetic, epigenetic, and immunological factors, have generated a spectacular improvement over the past decades, securing the path toward a specific and targeted immunotherapeutic approach and delayed progression to inflammatory arthritis. This review aimed to offer insight into various domains that underline the pathogenesis of psoriasis and how they influence disease development and evolution. The pathogenesis mechanism of psoriasis is multifaceted and involves an interplay of cellular and humoral immunity, which affects susceptible microbiota and the genetic background. An in-depth understanding of the role of pathogenic factors forms the basis for developing novel and individualized therapeutic targets that can improve disease management.

## 1. Introduction

Psoriasis is a chronic recurrent inflammatory autoimmune pathology with a significant genetic component and several interferences of immune cells and their cytokines [1,2]. It has a worldwide prevalence of about 2-3% of the general population, with variabilities depending on the skin phenotype, and it is lower in Asia or Africa and higher among Scandinavian populations [3,4]. An increased incidence is recorded in adults, equally distributed between men and women, with an onset in early ages for female patients, especially in cases of positive family history [5]. Clinically featured by several types, most cases correspond to psoriasis vulgaris or plaque-type psoriasis, pathologically characterized by an abnormal proliferation and differentiation of keratinocytes [6].

The severity of the psoriatic disease arises not only from its progressive evolution and association with psoriatic arthritis but also via its several comorbidities that share a common pathogenesis [7].

Contemporary research studies focused on the current understanding of psoriasis pathogenesis have led to fundamental insights that allow for a highly efficient targeted therapy that significantly impacts a patient’s quality of life [8].

The complex orchestration of psoriasis pathogenesis is related to the synergic effect of immune cells, polygenic alterations, autoantigens, and several external factors [9]. According to current scientific investigations, psoriasis is a T-cell-mediated pathology with an excessive production of IL-17 due to IL-23 stimulation. The major act of the IL-23/IL-17 axis, strongly influencing the inflammatory pattern established during disease activity, is visible as a continuous perpetuation of the pro-inflammatory response and keratinocyte activation and proliferation that lead to the development of psoriatic lesions [9,10].

Among the several factors involved in its complex etiology, dysbiosis, due to genetic or external stimulus, can induce a burst of pro-inflammatory consequences, with both the cutaneous and gut microbiome getting involved in the psoriasis pathogenic process [11]. With its role as both a physical and immune barrier toward external injuries, the skin has an extensive microbiome that can constitute the target of multiple factors, leading to its dysregulation and gaps in immunoregulatory processes [12]. The relevance of the gut microbiome is established by the interrelation between different components of both innate and adaptive immune systems, including the significant role of T cells [13].

Cutting-edge research studies and comprehensive insights into psoriasis pathogenesis fostering novel genetic, epigenetic, and immunological factors have generated a spectacular improvement over the past decades, securing the path toward a specific and targeted immunotherapeutic approach and delayed progression to inflammatory arthritis [8]. The breakthrough in therapeutic approaches has enabled the development of targeted agents, which are highly efficient, with fewer side effects and increased adherence from the patients. Although remarkable progress has been made in unrevealing psoriasis pathogenesis and improving disease management, the therapeutic area is still challenging, as prospective research remains imperative. 

This review aimed to offer insight into various domains that underline the pathogenesis of psoriasis and how they influence disease development and evolution. This review provides an in-depth description of the mechanisms involved in the pathogenesis of psoriasis through a holistic approach. Several domains are elaborated based on the evidence from the literature, and they reflect the complex nature of psoriasis and how different components interact during disease development and progression. The interplay between multiple pathogenic components explains the heterogeneity of clinical manifestations and offers the groundwork for research on advancing emergent therapies.

## 2. Genetic Disturbances in Psoriasis

Genome-wide association studies (GWASs) along with next-generation sequencing (NSG) analysis brought forth a coherent view of psoriasis pathogenic pathways, strongly impacted by genomic alterations (Single nucleotide polymorphisms (SNPs), copy number variations (CNVs), and epigenetic changes, and further possible therapeutic options [14]. Over the last years, the paramount amount of data on the genetic characterization of specific pathologies has been generated by employing the GWAS approach [14,15,16,17]. GWAS has identified ten loci, with the most proven association from PSORS1 to PSORS10. The recorded data indicate an extensive interference of several genes, including the ones associated with the functionality of the skin barrier, adaptive-immunity-related genes, especially CD8+ T and CD4+ T lymphocytes, and genes that belong to the histocompatibility complex (MHC) region on chromosome 6 [17]. 

Approximately one-third of psoriasis’s genetic impact on psoriasis development is associated with the MHC region, with genetic loci located on chromosome 6. The most eloquent genetic factor of psoriasis, PSORS1, was identified in the MHC I site, where human leukocyte antigen (HLA)-Cw-6 was considered the PSORS1 risk variant. PSORS1 lies within the scope of a 300 kb critical region in the MHC, where research studies have identified about fifteen genes strongly related to psoriasis. The recorded data indicate that the HLA-C is the most likely PSORS1 gene [18]. The risk allele HLACw0602*, present in up to 10-15% of the general population, inputs a 20-fold higher risk for developing psoriasis and is proven to be present in about 60% of psoriasis patients. It has been identified predominantly in young subjects with a familial history of psoriasis, where various infections, predominantly *Streptococcal* ones, trigger the first lesions [19].

Moreover, subjects with homozygous HLA-Cw0602* allele manifested a five times higher psoriasis risk than heterozygous ones [20]. A recent study published in 2021 that included 124 patients with psoriasis that aimed to establish the possible correlation between HLA-Cw0602* positivity and response to treatment showed a lower response for the cases with this risk allele [21]. 

Clinical tests identified PSORS2 on chromosome 17q and described CARD14 (caspase recruitment domain-containing protein 14) as being associated with psoriasis. CARD14, highly expressed in keratinocytes, shows a subsequent activating action on NF-κB and an increased release of pro-inflammatory cytokines [22,23,24]. Another two genes, SLC9A3R1 and NAT9, associated with psoriasis, were found on chromosome 17q and are responsible for RUNX1 transcription factor binding, directly correlated to T cells’ functionality [25,26]. 

PSORS4 resides on chromosome 1q21, and it has been associated with the presence of LCE genes (late cornified envelope genes), as the deletion of LCE3B and LCE3C is highly related to psoriasis. The expression of this protein is significantly increased in psoriatic lesions [27,28]. 

A significant impact on the development of both psoriasis and psoriatic arthritis is due to rs4349859 SNP located in the HLA-B gene, linked to HLA-B27 [29]. The presence of HLA-B27 predicts the development of specific future PsA characteristics, such as dactylitis, axial involvement, and also a more severe evolution of the disease, especially in early-onset cases; it was identified, reversely, that the presence of the HLA-B57 leads to a decreased risk of developing psoriasis [30]. 

Although research studies have validated the genetic impact on psoriasis, they have recorded no evidence of a single genetic variant to initiate the disease’s appearance; the interference of several genetic mutations is required, along with other pathogenic pathways.

## 3. Microbiome

With its role as both a physical and immune barrier toward external injuries, the skin has an extensive microbiome that can constitute the target of multiple factors, leading to its dysregulation and gaps in immunoregulatory processes [31,32,33]. The largest organ in the human body hosts several bacteria, viruses, and fungi, all contributing to maintaining a balance in the immune system. The range and distribution of skin microbiota are highly different depending on the site and unique for each individual, dependent on external conditions, associated comorbidities, sex, age, or hygiene level, with marked importance for different pathologies’ development [34]. 

Multiple studies have reported changes in the skin microbiome for different pathologies, including psoriasis, and the analysis of specimens from both lesions and regular areas, along with probes from controls, have enlightened the essential data. In 2008, Gao et al. reported that *Firmicutes* was the most plentiful phylum populating psoriatic skin and *Actinobacteria* in unaltered samples from psoriasis patients and healthy individuals [35]. A study published by Fahlén et al. revealed that there were three significant microorganisms present both in normal and psoriatic skin, *Firmicutes*, *Proteobacteria*, and *Actinobacteria*, as well as a significantly higher level of *Proteobacteria* in psoriasis compared to controls. Another important observation of the report indicates the lower level of *Staphylococci* and *Propionibacteria* in psoriasis patients compared to healthy individuals [36].

In 2013, Alekseyenko et al. analyzed lesioned and non-lesioned skin samples of 54 psoriasis patients and 37 controls and stated the relationship between *Firmicutes* and *Actinobacteria*-rich cutaneous-type and psoriasis. Also, an essential conclusion of the report that underlines the importance of skin microbiome analysis and establishing specific biomarkers endorsed the presence of significant skin genera, including *Corynebacterium*, *Proponibacterium*, *Staphylococcus*, and *Streptococcus*, both in lesions as well as in healthy skin samples of patients with psoriasis, along with a decrease of other taxa, including *Cupriavidus*, *Methylobacterium*, and *Schlegella* [37]. 

In 2018, Fyhrquist et al. analyzed the skin microbial population related to patterns of cutaneous gene expression in patients with atopic dermatitis or psoriasis. They discovered only weak relationships between potential pathogens and the expression of host transcripts in patients with psoriasis, with an important role attributed to *Corynebacterium*, which can perform a regulatory role that is potentially protective [38]. Later, Quan et al., in a report published in 2020, showed that there is certainly an increased difference between samples from lesions and unaffected skin, as well as controls, with *Propionibacterium* and *Corynebacterium* dominating the probes with psoriasis. The results also showed a positive correlation between the lesions’ severity and *Corynebacterium* species’ presence. The severity of the lesions, quantified using PASI (Psoriasis Disease Activity Index), was confirmed to be directly related to the presence of *Corynebacterium* species [39]. 

Chang et al. performed an analysis of skin bacterial species that revealed significant inequalities between the psoriasis-associated and healthy skin microbiota, arguing that a decrease of certain regulatory species, such as *Staphylococcus epidermidis* and *Propionibacterium acnes*, may prompt an increased settlement of *Staphylococcus aureus*. This event subsequently leads to an enhanced cutaneous inflammatory process via the Th17 axis [40]. 

Another relevant study, published by Tett et al., using high-resolution shotgun metagenomics to characterize the microbiome of psoriatic and unaltered skin from 28 patients, demonstrated that members of the genus *Staphylococcus* are significantly more abundant on diseased skin compared to unaffected skin. The results also showed the presence of different other bacteria and *Malassezia* spp., an abundant fungal type of skin [41]. 

Although the part of *Malassezia*, a lipophilic and lipid-dependent commensal fungus, is not entirely understood in psoriasis, Rivolta et al. described it as early as 1873 from a psoriasis lesion [42]. The authors found T cells reactive to *Malassezia* yeast and antibodies against *Malassezia* [43] in lesioned skin but not in normal subjects. Also, *M. Globosa* was the most commonly isolated species during psoriasis exacerbation [44]. It is also of utmost importance that the increase in LL-37, a cathelicidin antimicrobial peptide with a significant role in IFN-α production, initiation, and perpetuation of psoriatic lesions, can occur after *Malassezia* colonization of the skin [45]. 

Smoking has been recorded as an additional supporting part when analyzing the skin microbiome in patients with psoriasis, as it is a well-known promoter of several inflammatory autoimmune conditions [46]. 

The colon is the leading site of microorganism distribution, followed by the skin, with a diversity of microbiomes settled from the first years of life and influenced by genetics, lifestyle, or use of certain medications [38,47,48]. Disruptions in the gut microbiome may promote an increased risk of metabolic and autoimmune conditions, with the potential to initiate or sustain an inflammatory status, including psoriasis and its well-described comorbidities [49,50]. Despite several studies and promoted advancements in sequencing technologies, the limited number of patients included led to slight differences in the reports recorded, with the benefit of providing important up-to-date information and outlining future research directions. 

The gut microbiome integrates an extensive number of bacterial types, represented mainly by *Actynobacteria*, *Bacteroides*, *Firmicutes*, *Fusobacteria*, *Proteobacteria*, and *Verrucomicrobia*, as well as viruses, fungi, protozoa, or Archaea, maintaining a symbiotic status with the host highly influenced by age, genetics, dietary manners, and environmental, external factors [13,51,52,53]. Changes in the local intestinal microbiome can interact with skin homeostasis by influencing systemic immunomodulatory mechanisms [52]. 

Several reports have concluded that the changes seen in patients with psoriasis are relatively similar to those identified in patients with inflammatory bowel diseases, with an exuberance of *Actinobacteria* and *Firmicutes*, conjunctively to the *Firmicutes-to-Bacteroides* ratio, representing the model of the altered gut epithelial barrier [49,53,54,55,56]. 

The study of intestinal microbiota profiling reported by Chen et al. in 2018 evidenced that *Ruminococcus* and *Megasphaera*, of the phylum *Firmicutes*, were the top two genera of discriminant abundance in psoriasis, and they also noted decreased abundance of the phylum *Bacteroidetes* [49]. Analyzing the samples from 35 patients with psoriasis and 27 controls, Huang et al. reported that the relative abundances of *Firmicutes* and *Bacteroidetes* showed inversions at the phylum level. Sixteen kinds of phylotypes at the genus level indicated essential distinctions. Furthermore, *Proteobacteria* and *Actinobacteria* were also underrepresented in psoriasis patients [55]. 

Shapiro et al. documented a significant increase in the *Firmicutes* and *Actinobacteria* phyla compared to controls. At the species level, the psoriatic patients presented significant increases of *Ruminoccocus gnavus*, *Dorea formicigenerans*, and *Collinsella aerofaciens*. At the same time, *Prevotella copri* and *Parabacteroides distasonis* were significantly lower compared to controls [54]. The study by Masallat et al. reported statistically significant differences in the *Firmicutes* and *Bacteroidetes* ratio, which was directly associated with the PASI score. *Actinobacteria* manifested at a high level for controls. The results suggest that the differences in the gut microbiome are the source for counteracting and inducing inflammation, respectively, and therefore inducing psoriasis [53]. 

Another study, performed on 52 patients with psoriasis, revealed that the microbiome obtained was marked by increased *Faecalibacterium* and a decrease of *Bacteroides*, with higher values of *Akkermansia* and *Ruminococcus genra* compared to controls [57]. Discordant with these results, Scher et al. obtained a significant reduction in *Akkermansia*, *Ruminococcus*, and *Pseudobutyrivibrio* in the analyzed samples [56]. 

Several scientific publications have reported other types of bacteria found in increased levels in patients with psoriasis, mentioning *Bacillus*, *Subdoligranum*, *Slackia*, *Christensenella*, *Dorea*, *Coprococcus*, *Collinsella*, *Blautia*, *Enterococcus*, or *Lactocococcus*, as well as others exhibited in relatively low concentrations, such as *Allobaculum*, *Alistipes*, *Barnsiella*, *Gordonibacter*, or *Paraprevotella* [48,56,58]. 

Regarding individual species, some studies report an increased level for *Escherichia coli*, *Clostridium citroniae*, *Collinsella aerofaciens*, and *Dorea formicigenerans* [53,54,59].

Besides the stated role of different bacterial species, viral infections, including human papillomavirus or fungi, such as *Candida albicans* or *Malassezia*, have been interrelated with psoriasis. Also, *Candida albicans* activates dendritic cells via its ligand, beta-glucan, thus inducing the production of IL-36α with a further development of the psoriasis phenotype [60].

The interrelation between the innate and adaptive immune systems’ components establishes the gut microbiome’s relevance in psoriasis’s pathogenesis [52,61,62,63,64,65,66]. The leading role of the gut–skin axis in psoriasis is endorsed by T cells through the imbalance between Treg and Th17 cells [67,68]. Research findings suggest that the absence of microbiota, or its change, decreases the pro-inflammatory T cell response and further decreases the severity of cutaneous inflammation. Further studies support this perspective by describing the ability of commensal bacteria to modulate T cell development. An increased epithelial permeability after chronic inflammation derived from gut dysbiosis is one of the underlying mechanisms of skin impairment. 

The most relevant studies on the skin and gut microbiome in psoriasis are presented in Table 1.

Metabolic disturbances indicate another pathway for a systemic inflammatory state, activating several pattern recognition receptors on epithelial cells. Effector T cells activate due to the altered integrity of the mucosal cells and increased permeability. The bidirectional relationship between gut microbiota and mucosal epithelial cells, directly impacting the protective and functional status, is driven by the specific metabolites or immune-modulating factors, among which it is worth mentioning short chain fatty acids (SCFAs), retinoic acid, and polysaccharide A, as a result of the fermentation of non-digestible substrates, complex polysaccharides, and indigestible oligosaccharides by colonic microorganisms [54,63]. SCFAs modulate glucose and lipid metabolism [69], maintain gut mucosal integrity [70], and regulate the immune system and inflammatory responses [71]. Different mechanisms mediate all of these actions, including specific G-protein-coupled receptor family (GPCR) and epigenetic effects [72,73,74,75]. 

Disruptions in the gut microbiome balance and the further activation of T cells via interactions with pattern recognition receptors and Toll-like receptors trigger an inflammatory process and induce autoimmune conditions, such as rheumatoid arthritis [76], inflammatory bowel disease [77], systemic lupus erythematosus [78], multiple sclerosis [79], psoriasis [52,61,62,63,64,65,66,67,68], as well as other skin alterations, such as atopic dermatitis [80] and vitiligo [81].

## 4. Immune Cells

### 4.1. Keratinocytes (KCs)

Psoriasis is a pathology characterized by hyperproliferation and the disturbed differentiation of KCs, cells with both structural and immune roles that interfere in the early stages of the disease and support the maintenance of chronic inflammation [82]. They constitute the main cellular population altered in psoriasis, as they express receptors for all types of cytokines involved in the complex pathogenesis of the disease [83,84,85].

Consequent to dermal injury, one can observe an increased secretion of antimicrobial peptides, cathelicidin, LL-37, S100 proteins, and β-defensins from keratinocytes, promoting the activation of pDCs and the release of IFN-α, IFN-γ, TNF-α, and IL-1β [86,87].

Besides the stated role in the early stages of the disease, they continue to perpetuate the inflammatory process during disease evolution. After activation, they continuously produce chemokines in high amounts (CXCL1/2/3, CXCL8, CXCL9/10/11, CCL2, and CCL20), recruit leucocytes or antimicrobial peptides, and stimulate the inflammatory process. In addition, fibroblasts and endothelial cells significantly contribute to tissue restructuring, consecutive to their interaction with Th17 cells [88,89,90].

### 4.2. T Cells

It is well-established that psoriasis is a T-cell-mediated disease, considering that effector T cells transmigrate from the lymph nodes into the systemic circulation and derive from the injured skin sites [91,92]. All types of T cells, CD4+ T cells (Th), CD8+ T cells (Tc), and Treg cells are involved in the initiation and perpetuation of psoriatic lesions [93]. Pro-inflammatory cytokines IL-12, IL-23, and TNF, released by dendritic cells (DCs) and activated macrophages, prompt the activity of Th cells (Th1, Th17, Th22) and promote epidermal hyperproliferation with an inhibition of apoptosis [94,95]. Concomitant, IL-17 and TNFα stimulate keratinocytes to release pro-inflammatory cytokines. In the early course of the disease, psoriasis patients express high levels of IFN-γ, which shifted toward IL-10 secretion in chronic patients, suggesting a possible shift from Th1 to Th2 response as an adaptation of the immune system to down-regulate inflammatory Th1 response [93].

Another cytokine contributing to psoriasis pathogenesis is Th9, with a majority of the memory Th9 cells being either skin-tropic or skin-resident. IL-9, released by Th9, is essential for the production of IFN-γ, IL-17, and IL-13 by skin tropic T cells to the maximal level [96]. Treg cells, known to conserve immune stability, are found at a low level in psoriasis. Upon an increased pro-inflammatory cytokine environment, psoriatic Tregs behave like Th17 cells and cannot suppress the T effector activation [97,98].

### 4.3. B Cells

*B Cells* are known to be regulators of the pathogenesis in several autoimmune diseases by producing autoantibodies and antigen presentation. Thus, research studies have not extensively studied *B Cells* as lymphocyte T in patients with psoriasis, and their input on psoriasis genesis and perpetuation is not completely clear [99]. Resident cutaneous B cells contribute to skin homeostasis and regulate reparatory processes and the local microbiome. Also, experimental studies have proved that in areas of inflamed skin, there is a high quantity of Il-6, IL-4, GM-CSF, or IFN-γ [100,101,102,103].

### 4.4. Dendritic Cells (DCs)

Several types of DCs (plasmacytoid DCs (pDCs), conventional DCs (cDCs), and Langerhans cells (LCs)) are involved in the pathogenesis of psoriasis, with numerous reports demonstrating the presence of pDCs and cDCs in the lesional skin samples of these patients [104]. Consecutive to different external stimulus, DCs activate and commute to professional antigen-presenting cells (APCs), with subsequent interaction with naive T cells and production of pro-inflammatory cytokines, TNF-α, IL-23, IL-12, and IL-6 that furthermore activate an inflammatory reaction, keratinocyte proliferation, and recruitment of neutrophils [105,106].

A subset of inflammatory DCs, TIP-DCs, secrete TNF and inducible nitric oxide synthase (iNOS) with a pro-inflammatory response in patients with psoriasis [107,108,109].

LCs, cells with an essential contribution to local skin immunity, show a debatable role in psoriasis pathogenesis. Experimental studies have shown divergent results, with reports revealing that they stimulate the secretion of multiple inflammatory cytokines and induce the proliferation of IL-17A-producing γδ T cells. Others suggest that they may exert an anti-inflammatory role in psoriasis by increasing the production of IL-10 (or IL-23 in case of LC depletion) [110,111,112].

### 4.5. Neutrophils

Excessive storage of neutrophils in psoriatic lesions is a typical disease feature, as they infiltrate the dermis in early stages and subsequently migrate to the epidermis [113]. After neutrophil activation, increased levels of reactive oxygen species (ROS) are released into circulation, with DC stimulation, T cell stimulation, and an altered balance between Th1 and Th2, with the final result being represented by keratinocyte proliferation and increased angiogenesis [114,115,116]. Secondary to excessive ROS production, LL-37 is released, with a production of IFN-alfa by pDCs or ILP6, IL-12, and IL-23 by mDCs [117,118]. In addition to Th17 cells, neutrophils represent a category of cells that can contribute to IL-17A production and the perpetuation of psoriasis development. Moreover, keratinocytes activate neutrophils to produce pro-inflammatory cytokines and augment dermal inflammation [119,120].

### 4.6. Macrophages

Antigen-presenting cells derived from monocytes are shown to be present in psoriatic lesions, with TNF secretion upon activation and regulation of angiogenesis by releasing VEGF [121,122].

### 4.7. Natural Killer (NK) Cells

These are present in the lesional skin of psoriasis patients; they exhibit reduced degranulation and produce lower levels of the pro-inflammatory cytokines IFN-γ and TNF-α [86].

## 5. Cytokines

### 5.1. Th17 Cytokines

One of the first studies describing cytokines in the dermal samples of psoriasis patients, published in 1994 by Schlaak et al., revealed that Th1-related cytokines prevailed in the analyzed probes, with a few Th cells releasing Th-2-related cytokines [123]. Several further reports confirmed these results and raised the hypothesis that psoriasis can be defined as a Th1-mediated disease.

It is currently well-established that the pathophysiology of the disease comprises a vast network of immune cells, which, along with their cytokines, initiate the inflammatory response [124]. The Th17 cytokine family is the major effector in the pathogenesis of psoriatic disease, and it strongly influences the inflammatory pattern established during disease activity [125,126]. In addition, the vast network of cells that orchestrates the pathophysiology makes psoriasis complex to study. 

IL-23, which is part of the IL-12 family, is produced by both resident [blood dendritic cell antigen (BDCA-1)/CD1c+] and inflammatory myeloid DCs (CD11c+ BDCA-1/CD1c–), as well as macrophages (CD163+) in psoriasis. It is responsible for the activation, differentiation, and proliferation of Th17 cells that further initiate the release of acting cytokines IL-17A or IL-21, with effects on neutrophils’ draft [127,128,129,130,131,132]. In psoriasis patients, the IL-23 pathway is activated and characterized by high-level production of IL-23 by DCs and keratinocytes and increased numbers of Th17 cells [133].

IL-17 is an acknowledged cytokine produced mainly, but not exclusively, by Th17 cells [134]. Besides Th1 cells, mast cells, γδ T cells, αβ T cells, and innate lymphoid cells represent primary sources of IL-17 production [135,136]. From the six subtypes of the IL-17 family, IL-17A and IL-17F have been proven to be found both in psoriasis plaques as well as in unaltered skin, with IL-17A playing a central part in the pathogenesis of the disease [134]. The cytokines mentioned above are expressed together and act synergistically on the upregulation of inflammatory markers.

There are several mechanisms with intricate casting in psoriasis induction by which IL-17 exerts its action on immune cells, keratinocytes, and fibroblasts, inducing the release of inflammatory mediators, including cytokines, IL-6, IL-1β, TNF, and granulocyte–macrophage colony-stimulating factor (GM-CSF), chemokines (CXCL1, CXCL2, CCL20, and CXCL8), matrix metalloproteinases (MMPs), antimicrobial peptides (AMPs; LL37, S100s, β-defensin), and complements [137,138,139]. Thus, it initiates the recruitment and activation of neutrophils, lymphocytes, and myeloid cells, with local dermal inflammation, an effect developed by the synergic effects of IL-23 and possibly IL-36. Increased IL-17, produced by CD4+ Th17 cells, can also be detected in the synovial fluid of patients with psoriasis. The effect of IL-17 goes beyond epithelial cells, which are also identified in endothelial cells, with a pro-coagulant activation [139,140].

IL-21 is a cytokine still under current investigations regarding its entire part in psoriasis, with high amounts of IL-21 receptor (IL-21R) revealed in keratinocytes. It represents a future therapeutic target as research studies reported it significantly affecting Th17 proliferation, mediated by regulatory T cells (Treg) [141,142].

### 5.2. Th1 Cytokines

IL-12 is an activator of Th1 differentiation and proliferation, with consequent production of TNF-β, IFN-γ, and IL-2, found to be increased in patients with psoriasis [143]. A significant implication has been reported in psoriatic arthritis (PsA), where high levels of its soluble receptor (IL-12R) directly correlate with the degree of cutaneous involvement [144,145]. IL-12 inhibitory agents prove their efficacy as they diminish the expression of IFN-γ and inhibit the release of IL-8 by keratinocytes; additionally, experimental studies have shown a protective role of this cytokine in the IL-23’Th17 pathway [131].

### 5.3. IL-8

Macrophages, epithelial cells, endothelial cells, and respiratory smooth muscles release a cytokine that is a marker of inflammation throughout neutrophils’ attraction. Keratinocyte production of IL-8 is stimulated by visfatin, an adipokine produced by leukocytes and adipose tissue, under TNF-α stimulation [146].

### 5.4. IL-22

IL-22 is a member of the IL-10 family that impairs keratinocyte proliferation. It is associated with psoriasis progression when detected in high levels. It is secreted by NK cells, Th17 or Th22, with a marked input on the keratinocyte cycle during regenerative processes [147]. Notably, the study by Kagami et al. showed that IL-22 deficiency caused a significant decrease in epidermal acanthosis and dermal inflammation induced by IL-23 [148]. In addition, the study published by Van Belle et al. showed that IL-22 has not only a significant part in the development of pustules and acanthosis but also in neutrophil infiltration in a mouse model triggered by the Toll-like receptor (TLR) 7/8 agonist imiquimod [149].

### 5.5. IL-1β

IL-1β is a cytokine highly expressed in psoriasis samples, and two genetic variants, rs16944 and rs2853550, were proven to be associated with late initiation of the disease and a slower progression [150].

### 5.6. IL-6

IL-6 is expressed in high levels in the psoriatic lesion. It is a pleiotropic pro-inflammatory cytokine that is produced by a variety of cells, such as fibroblasts, macrophages, endothelial cells, and KCs, in response to a variety of stimuli, which include other cytokines, such as IL-1, TNF-α, and PDGF. IL-6 stimulates the proliferation of human KCs. Anti-IL-6 therapies, effective for rheumatoid arthritis, are either ineffective for psoriasis or can induce new-onset psoriasis-like disease [151].

### 5.7. TNF-α

TNF-α has been identified as a critical cytokine mediating cutaneous inflammation in the pathogenesis of psoriasis [152]. TNF is a homotrimer cytokine released mainly by immune and epithelial cells. It exerts its effects by binding to two receptors: TNFR1/p55 (expressed ubiquitously and constitutively) and TNFR2/p75 (expressed only on immune, endothelial, and neuronal cells, which are inducible).

Over the years, several studies have reported increased levels of TNF, TNFR1, and TNFR2 observed in psoriatic lesions, which are produced by various cellular populations involved in the pathophysiology of psoriasis, such as keratinocytes, dendritic cells (DCs), and NKT, Th1, Th17, and Th22 cells. TNF induces immune and inflammatory responses orchestrated by keratinocytes and tissue remodeling, cell motility, cell cycling, and apoptosis [153,154]. Additionally, activated keratinocytes produce many chemokines responsible for recruiting neutrophils, macrophages, and skin-specific memory T cells. These observations underline that TNF might be involved in psoriasis’s initial and chronic phases [155].

### 5.8. T Reg Cells

T Reg Cells that express FOXP3 constitute significant regulators of the immune system and the inflammatory response. They inhibit the action of various immune cellular populations either directly or by releasing cytokines, such as IL-10 or TGF-β, that act in a suppressing manner. The activity of T reg cells is probably impaired in psoriasis, as data sustain that STAT3, activated by pro-inflammatory cytokines, inhibits T reg.

IL-10, an anti-inflammatory cytokine produced by regulatory T cells, reported as a significant mediator in psoriasis, proved to be an essential inhibitor for pro-inflammatory T cell responses and keratinocyte inflammatory markers. Asadullah et al. reported that the levels of mRNA and the cytokine itself decreased significantly in patients with psoriasis compared to other dermatologic disorders. Additional reported information was that administering recombinant IL-10 for 30 days diminished the PASI score and Th-1 cytokine levels [156].

**Figure 1:**
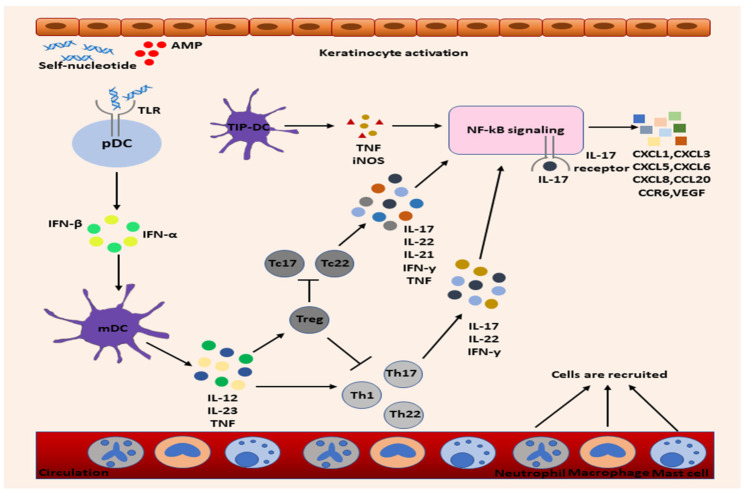
Immune cells in psoriasis. Consequent to external injuries, keratinocytes release AMPs, form complexes with self-nucleotides, and further attach to TLR and secrete IFN-α/β. Activated mDCs release pro-inflammatory cytokines, prompt the activity and differentiation of T cells, and control the inflammatory events. Activated keratinocytes continuously produce chemokines in high amounts, recruit leucocytes, and amplify the inflammatory process. AMP—antimicrobial peptide; iNOS—inducible nitric oxide synthase; mDC—myeloid dendritic cells; pDC—plasmacytoid dendritic cells; TLR—Toll-like receptors; Treg—T regulatory cells.

The interplay between immune cells and their part in the complex inflammatory process that characterizes psoriasis is described in Figure 1.

## 6. Intracellular Signaling Pathways

### 6.1. Janus Kinase-Signal Transducer and Activator of Transcription (JAK/STAT Pathway)

JAK is a member of the tyrosine kinase (TYK) family, which comprises four members, JAK1, JAK2, JAK3, and TYK2, with various expressions depending on the cellular type [157]. The development of various inflammatory autoimmune diseases depends on JAKs activation and phosphorylation of STATs throughout several pro-inflammatory cytokines.

**Figure 2:**
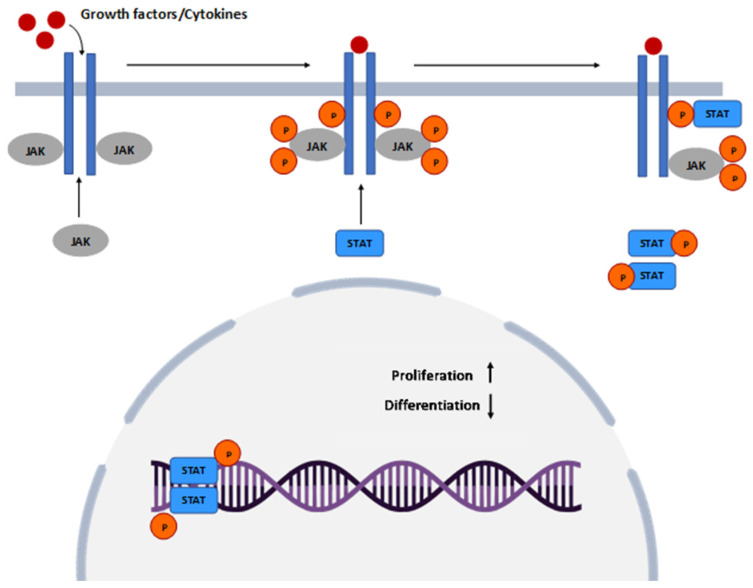
JAK–STAT signaling pathway in psoriasis. Consequent to cytokines and growth factors binding to receptors, JAK is recruited and phosphorylated, an event that leads to receptor tyrosine phosphorylation and the development of a binding site for STAT. After STAT phosphorylation, the receptor unlocks and forms a dimer that penetrates the nucleus, attaches to DNA, and directs transcription with further increased proliferation and decreased differentiation of keratinocytes. JAK—Janus Kinase; STAT—Signal Transducer and Activator of Transcription.

The activation of the JAK/STAT pathway in psoriasis is described in Figure 2. Although the pathogenesis of psoriasis comprises multiple types of cytokines, the central role is attributed to IL-23 and Th17, which are directly linked to the JAK/STAT pathway [158]. An increased expression of STAT1, directly related to JAK1/JAK2, determines the activation of IFN-α/β and IFN-γ. IFN-γ and IL-12, through a TYK2-dependent mechanism, have significant input on keratinocyte stimulation and promote psoriatic inflammation [159]. The study conducted by Nada et al. highlighted a vital outcome that reported a positive correlation between JAK1 and the PASI score and a significantly higher level compared to controls [160].

Other reports performed on mouse models of psoriasis have concluded that TYK2 can slow the progression of psoriasis. STAT3, a central regulator for Th17 differentiation via IL-23 activation, is overexpressed in psoriasis skin samples, and it constitutes a key regulator for keratinocytes [159].

Similarly, IL-22 stimulates keratinocyte proliferation and inhibits their differentiation consecutive to STAT3 activation [161,162].

### 6.2. A3 Adenosine Signaling Pathway

Adenosine, generated by ATP catabolism, acts as a suppressive metabolite with immunomodulatory and anti-inflammatory functions enabled through four receptor subtypes: A1, A2A, A2B, and A3 [163]. A3 receptors are expressed on all types of immune cells and exert an anti-inflammatory action against LPS-induced cytokine release, thus enabling the development of various selective A3 receptor ligands. Evidence of the role of A3Ars in modulating immune cell function and positive results obtained in rheumatoid arthritis studies have led to the development of a specific inhibitor, CF101 (piclidenoson), for treating psoriasis. It has shown positive results with a reduction in the PASI score. In addition, it lowers the expression of pro-inflammatory markers, like TNF- α, IL-17, IL-23, and phosphoinositide-3-kinase (PI3K) [164].

### 6.3. WNT

The WNT signaling pathway is essential in cell growth, differentiation, and migration. In psoriasis, WNT signaling generates an overexpression of WNT5A, with studies showing increased levels in affected areas and increased expression of its receptor proteins FZD2 and FZD5 [165]. Data suggest that the epidermis is one of the primary sources of WNT5A in psoriatic lesions, affecting inflammatory responses of human mononuclear cells and blood vessel proliferation [166].

### 6.4. NF-κB Signaling

The transcription factor NF-κB directs several processes, including inflammation, proliferation, immunological actions, apoptosis, and differentiation. Compared to standard samples, the psoriatic derma presents high levels of active phosphorylated NF-κB. It influences psoriasis pathogenesis by stimulating TGF expression, VEGF induction, and angiogenesis; it also binds to keratinocytes, with consequent cytokine production and chemokine activation [167].

## 7. Immunotherapy in Psoriasis

Cutting-edge research studies and comprehensive insights into psoriasis pathogenesis fostering novel genetic, epigenetic, and immunological factors have generated a spectacular improvement over the past decades, securing the path toward a specific and targeted immunotherapeutic approach and delayed progression to inflammatory arthritis [168,169]. Current therapeutic recommendations focus on implementing treat-to-target (T2T) strategies and aim for increased patient adherence, paramount to achieving therapeutic success. State-of-the-art biologicals and highly developed targeted synthetic drugs effectively influence the critical agents of the autoimmune system, including TNF-α, interleukin (IL)-12/23, IL-17A, or the JAK–STAT pathway [170,171].

### 7.1. TNF-α Inhibitors 

TNF-α Inhibitors were the first biologics approved as a therapeutic option for both moderate and severe psoriasis and PsA. These agents fulfil their function as either soluble fusion proteins (*etanercept*) or monoclonal antibodies (*infliximab*, *adalimumab*, *certolizumab*). Although they have been proved to have sustained efficacy and a long-term safety profile, clinical studies have reported cases of severe adverse events, paradoxical reactions, and immunogenicity [171,172].

### 7.2. IL-23 Inhibitors

Pivotal research on the unfolding of psoriasis pathogenesis, specifically regarding Th helper 17 cells, has unlocked a new class of biologics that control different effectors along this pathway. Among these, IL-23 inhibitors constitute safe and highly efficient options for psoriasis and PsA patients [8]. They showed superior efficacy compared to previous therapies, including TNF-α inhibitors, with a favorable risk profile and marked input on patient quality of life [173]. *Ustekinumab* (a monoclonal antibody directed against the common p40 subunit of IL-12 and IL-23), *guselkumab* (the first human antibody against the p40 subunit of the IL-23 receptor), *risankizumab* (a humanized IgG monoclonal antibody that binds to the p19 subunit of IL-23 and inhibits its interaction with the IL-23 receptor), and tildrakizumab (a humanized IgG1 monoclonal antibody targeting IL-23 p19) are effective IL-23 inhibitors indicated for psoriasis [8,173]. *Mirikizumab*, a humanized IgG4 monoclonal antibody that targets the p19 subunit of IL-23, has been shown to be effective in phase III clinical trials, as its inhibitory mechanisms succeed in crossing psoriasis pathogenesis [8,174].

### 7.3. IL-17 Inhibitors 

IL-17 Inhibitors target either the IL-17 ligand or its receptor [10]. The first, *secukinumab*, is a humanized IgG1 monoclonal antibody that selectively binds to and neutralizes IL-17A with remarkable efficiency in psoriasis and PsA [175,176]. *Ixekizumab* is a humanized monoclonal IgG4 antibody with benefits similar to secukinumab [177]. *Brodalumab* targets IL-17 receptor α, with a high clinical response and a similar safety profile [178]. *Bimekizumab*, an inhibitor for both IL-17A and IL-17F, was shown to exert a faster and sustained effect compared to the IL-17 and IL-23 inhibitors used in prior studies, making it a promising new IL-17 inhibitor [179].

### 7.4. JAK Inhibitors 

JAK Inhibitors represent a novel class of immunosuppressive agents that act as signal transducers and activators of STAT proteins. Tofacitinib, the primary JAK inhibitor, targets JAK1-3, and, consistent and sustained remission achieved for PsA patients, proved effective for moderate-to-severe plaque psoriasis during several clinical studies [180]. *Baricitinib* is a JAK1 and JAK3 inhibitor that decreases the release of IFN-γ, IL-12, IL-17, and IL-23 [180,181]. *Ruxolitinib* is a selective JAK1 and JAK2 inhibitor, with consequent inhibition of STAT3 phosphorylation and Th17 cell apoptosis [182]. Although the JAK inhibitors mentioned above have proved to be highly effective in clinical trials, they are not currently approved, given the reported side effects and long-term safety concerns. Thus, specialists in the field have looked into additional safety analyses to further consolidate the results obtained [180]. Second-generation JAK inhibitor *deucravacitinib* is a highly selective TYK2 inhibitor that targets key inflammatory pathways involved in psoriasis pathogenesis. Having brought significant improvement to multiple efficacy measures applied in several clinical studies, current recommendations validate it for longer-term safety and tolerability [171,183].

### 7.5. RORγt Inhibitors 

RORγt Inhibitors are pharmacologic agents targeting retinoic-acid-receptor-related orphan receptor gamma-t (RORγt), a transcription factor essential for Th17 cells. They reveal significant potential as a new therapeutic approach by controlling the Th17-cell-mediated immune response [184].

### 7.6. Rho-Associated Protein Kinase 2 (ROCK2) Inhibitors

Rho-Associated Protein Kinase 2 (ROCK2) Inhibitors impact the inflammatory response and diminish the discharge of pro-inflammatory cytokines, arising as a promising choice for psoriasis treatment [185].

The breakthrough in therapeutic approaches has enabled the development of targeted agents, which are highly efficient, with fewer side effects and increased adherence from the patients. Although remarkable progress has been made in revealing psoriasis pathogenesis and improving disease management, the therapeutic area is still challenging, as prospective research remains imperative.

## 8. Conclusions

The pathogenesis mechanism of psoriasis is multifaceted and involves an interplay of cellular and humoral immunity, which affects susceptible microbiota and the genetic background. An in-depth understanding of the role of pathogenic factors forms the basis for developing novel and individualized therapeutic targets that can improve disease management.

## Figures and Tables

**Table 1:** Summary of most important reports on skin and gut microbiome in psoriasis.

Author(s)	Group Size	Analyzed Sample	Reported Dysbiosis
Gao et al. [35]	6 Ps patients 6 controls	Skin biopsy	↑ *Firmicutes* ↓ *Actinobacteria*, *Proteobacteria*
Fahlen et al. [36]	10 Ps patients 12 controls	Skin biopsy	↑ *Proteobacteria*, *Firmicutes*, *Actinobacteria* ↓ *Staphylococci*, *Propionibacteria*
Alekseyenko et al. [37]	54 Ps patients 37 controls	Skin swab	↑ *Firmicutes* ↑ *Actinobacteria* ↓ *Proteobacteria*
Fyhrquist et al. [38]	119 Ps patients 82 AD patients 115 controls	Skin swab	*↑ Corynebacterium kroppenstedtii*, *Corynebacterium simulans* *Finegoldia*, *Neisseriaceae* spp. ↓ *Lactobacili*, *Burkholderia* spp., *Cutinebacterium acnes*
Quan et al. [39]	27 Ps patients 19 controls	Skin swab	↑ *Corynebacterium* ↓ *Cutibacterium* (*Propionibacterium*)
Chang et al. [40]	28 Ps patients 26 controls	Skin swab	↑ *Staphylococcus aureus*, *Staphylococcus pettenkoferi*, *Proteobacteria* ↓ *Actinobacter*, *Staphylococcus epidermidis*, *Propionibacterium acnes*
Tett et al. [41]	28 Ps patients	Skin swab	↑ *Staphylococcus* genus ↑ *Malassezia* spp.
Chen et al. [49]	32 Ps patients 64 controls	Fecal sample	↑ *Firmicutes* ↓ *Bacteriodetes* ↑ *Firmicutes*/*Bacteriodetes* ratio
Huang et al. [55]	35 Ps patients 27 controls	Fecal sample	↓ *Firmicutes* ↑ *Bacteroidetes*
Shapiro et al. [54]	24 Ps patients 24 controls	Fecal sample	↑ *Firmicutes* ↓ *Bacteriodetes* ↑ *Firmicutes*/*Bacteriodetes* ratio ↑ *Actinobacteria*, *Blautia*, *Faecalibacterium*, *Ruminoccocus gnavus*, *Dorea formicigenerans*, *Collinsella aerofaciens* ↓ *Proteobacteria*, *Prevotella copri*, *Parabacteroides distasonis*
Masallat et al. [53]	45 Ps patients 45 controls	Fecal sample	↑ *Firmicutes*/*Bacteroidetes* ratio ↓ *Actinobacteria*
Codoñer et al. [57]	52 Ps patients	Fecal sample	↑ *Staphylococcus aureus*, *Staphylococcus pettenkoferi*, *Proteobacteria* ↓ *Actinobacter*, *Staphylococcus epidermidis*, *Propionibacterium acnes*
Scher et al. [56]	15 Ps patients 15 PsA patients 17 controls	Fecal sample	↓ *Akkermansia*, *Ruminococcus*, and *Pseudobutyrivibrio* in Ps and PsA ↓ *Coprococcus* in Ps ↓ *Bacteroidetes phylum* and *Coprobacillus* genus in Ps compared to PsA
Tan et al. [58]	14 Ps patients 14 controls	Fecal sample	↑ *Enterococcus*, *Bacteroides*, *Clostridium citroniae* ↓ *Akkermansia muciniphila*, *Verrucomicrobiaceae*
Eppinga et al. [59]	29 Ps patients 31 IBD patients 13 concomitant psoriasis and IBD 33 controls	Fecal sample	↑ *Escherichia coli* in Ps and IBD ↓ *Faecalibacterium prausnitzii* in Ps and IBD

Ps—psoriasis; AD—atopic dermatitis; PsA—psoriatic arthritis; IBD—inflammatory bowel disease; ↑—increased; ↓—decreased.

## Data Availability

Not applicable.
